# Treatment of Sore Nipples by Collodion

**Published:** 1849-09

**Authors:** 


*Treatment of sore Nipples By Collodion.*—

The following observations are quoted from Professor SIMPSON's paper on gun-cotton solution;—It has been proposed to use the etherial solution of gun-cotton for other purposes than the dressing and union of wounds—for example, as a substitute for the starch bandage in fractures; as an application and dressing to ulcers, &c. In abrasions, and slght injuries of the skin and fingers it forms an excellent and adhesive dressing. There is one extremely painful and unmanageable form of ulcer in which I applied it eight or ten days ago, at the maternity Hospital, with perfect success. I allude to fissures at the base of the nipple. Many practitioners know well the agonies some mothers undergo in consequence of this apparently slight disease; the ulcer or fissure being renewed and torn open with each application of the child. In two such cases I united the edges of the fissures, and covered them over with the solution of gun-cotton, making the layer pretty strong. It acted successful by maintaining the edges so firmly together that they were not again re-opened by the infant; the gun-cotton dressing was not, like other dressings, affected by the moisture of the child's mouth; and as a dressing, and at the same time by securing rest to the part, it allowed complete adhesion and cicatrization speedily to take place. I have applied it also repeatedly to ulcers of the cervix uteri and over various cutaneous eruptions. Its application relieves at once the smarting of slight burns.—*British Record.*

